# A retrospective comparative study of the clinical and radiological outcomes of intertrochanteric fractures treated with proximal femoral nail antirotation (PFN-A) and INTERTAN nail

**DOI:** 10.1371/journal.pone.0316954

**Published:** 2025-01-06

**Authors:** Hasan Orkun Varmış, Mehmet Yiğit Gökmen, İsmet Tan

**Affiliations:** 1 Department of Orthopedics and Traumatology, University of Health Sciences, Adana City Training and Research Hospital, Adana, Türkiye; 2 Çukurova University Faculty of Medicine, Department of Orthopedics and Traumatology, Adana, Türkiye; University Hospital Zurich, SWITZERLAND

## Abstract

**Background:**

This study aimed to analyze the files of patients treated using PFN-A or INTERTAN intramedullary nails to reveal additional superiorities or disadvantageous factors for selecting the better intramedullary fixation method in patients presenting with intertrochanteric femur fractures.

**Methods:**

In this retrospective study, the files of the patients who were operated on for intertrochanteric femur fractures using intramedullary fixation methods between September 2010 and June 2015 in the Orthopedics and Traumatology Clinic, Çukurova University Faculty of Medicine, were reviewed. The data including age, gender, chronic diseases, causes of fractures, fracture classification based on Arbeitsgemeinschaft für Osteosynthesefragen (AO), the nail type (long or short), the interval between trauma and surgery, duration of anesthesia and surgery, hospitalization duration, amount of blood transfusion, the Tip-Apex Distance (TAD) in postoperative radiographs, functional outcomes according to Harris Hip Score (HHS) and Western Ontario and McMaster Universities Osteoarthritis Index (WOMAC), postoperative complications, need for revision, and mortality was noted.

The data of the follow-up visits included physical examination findings in addition to the radiological assessment findings obtained using X-ray imaging involving the evaluation of the union in terms of the presence of infection, loss of reduction, implant failure, avascular necrosis, additional signs related to poor union formation, and the position data of the lag screw placement point according to Cleveland and Bosworth Quadrants.

**Results:**

The records showed that 194 files were available for analysis. The mean age of the patients included in the study was 70.9±16.4 years; 100 patients (51.5%) were male, and 94 (48.5%) were female. There were 76 patients (39%) in the PFN-A group and 118 patients (61%) in the INTERTAN group. Analysis of the mean Harris Hip Scores in both nail groups showed that only the mean scores at the 1st follow-up were statistically significantly different (P = 0.025).

**Conclusion:**

In conclusion, comparing the two nail types in terms of complication rates and clinical data, neither nail has proven superior to the other. Moreover, the temporary difference in the first follow-up HHS scores should not be considered an indicator of the overall functional outcome because long-term follow-up HHS scores did not differ.

## Introduction

Femoral intertrochanteric fractures are defined as extracapsular fractures that occur between the greater and lesser trochanter. Due to the vascular anatomy, the risk of nonunion and avascular necrosis in trochanteric region fractures is quite low [1]. The dramatic increase in average life years, mainly due to medical developments, has led to a rise in the occurrence of intertrochanteric femur fractures, which account for approximately 50% of all hip fractures occurring in the elderly population [1,2].

The reports on intertrochanteric femur fracture treatments in the elderly suggest that the primary goal should be returning the patient to ambulation or mobilization as soon as possible because complications such as pressure sores, pulmonary embolism, congestive heart failure, thrombophlebitis, and depression caused by prolonged bed rest in elderly patients lead to increased mortality and morbidity. Treatments that allow mobilization and accelerate the patient’s return to pre-fracture status by providing adequate reduction and firm fixation will prevent these significant complications [3].

On the other hand, orthopedic surgery may result in undesirable outcomes in special cases including the elderly, osteoporotic patients, and particularly in unstable intertrochanteric fractures. In addition, the success of surgery varies depending on the surgical fixation material, surgical technique, and bone quality [4].

Recent studies have shown that the dynamic hip screw (DHS) implant, formerly considered the most effective treatment for stable intertrochanteric fractures, was insufficient for stabilizing unstable fractures [5]. Yet the use of proximal femoral nails (PFN), with their shorter moment arm and closer position to the force line, was biomechanically superior to extramedullary implants and strongly recommended. The PFN was confirmed as advantageous with more efficient load transfer, reduced risk of mechanical failure by reducing the load on the implant and controlled fracture impaction, as well as addressing the common problem of medialization of the femoral diaphysis and recommended for use in all unstable fractures, pointing out that it allows patients to be mobilized with full load bearing immediately after surgery [6–8]. Furthermore, reports suggest that intramedullary fixation may be more advantageous than extramedullary fixation for patients due to a reduced likelihood of implant failure, reoperation, and greater functional ratings [9,10].

Recently, the Proximal Femoral Nail-Antirotation (PFN-A) and INTERTAN have gained popularity among intramedullary nail types for the treatment of intertrochanteric femoral fractures, with comparable advantages over each other depending on case characteristics. [11]. The PFN-A has a helical blade that provides both rotational and angular stability. The INTERTAN, on the other hand, has a unique design consisting of two cephalomedullary screws that interlock and allow controlled intraoperative linear compression of the intertrochanteric fracture. This design also ensures subsequent rotational stability of the head and neck fragment [9].

Some studies suggest that the INTERTAN system has superior biomechanical qualities and treatment outcomes [12], but other studies indicate that INTERTAN does not significantly improve functional recovery in patients [13]. There are few objective and comprehensive studies in the literature to guide clinicians on the optimal treatment, focusing on the comparison of mechanical performance between the use of PFN-A and INTERTAN in intertrochanteric femoral fractures.

This study aimed to analyze the files of patients treated using PFN-A or INTERTAN intramedullary nails to reveal additional superiorities or disadvantageous factors for selecting the better internal fixation method in patients presenting with such fractures.

## Methods

### Ethics statement

This study was approved by Çukurova University, Faculty of Medicine, Non-Interventional Clinical Research Ethics Committee, with decision number 3 of meeting number 22. Written informed consent was obtained from all the participants. The medical records database was accessed on December 20, 2015. The records were obtained anonymously; no patient name or personal information was included.

### Study design and data collection

In this retrospective study, the files of the patients who were operated on for intertrochanteric femur fractures using intramedullary fixation methods between September 2010 and June 2015 in the Orthopedics and Traumatology Clinic of Balcalı Hospital, Çukurova University Faculty of Medicine, were reviewed. The data including age, gender, chronic diseases, causes of fractures, fracture classification based on Arbeitsgemeinschaft für Osteosynthesefragen (AO), the interval between trauma and surgery, the nail type (long or short), the Tip-Apex Distance (TAD) in postoperative radiographs, screw placement point according to Cleveland and Bosworth Quadrants, duration of anesthesia and surgery, hospitalization duration, amount of blood transfusion, union rates, functional outcomes according to Harris Hip Score (HHS) and Western Ontario and McMaster Universities Osteoarthritis Index (WOMAC), postoperative complications, need for revision, and mortality was noted.

Patients with pathological fractures, concomitant fractures of another extremity, hip arthritis, lumbar disc herniation, or other conditions affecting hip function, patients who could not be reached for various reasons (change of contact information, death, etc.), patients with incomplete medical records and patients with missing follow-up data were excluded from the analysis. The remaining 194 patients were dichotomized into two groups: PFN-A and INTERTAN. The number of cases operated using the PFN-A (PFNA^®^, designed by AO) was 76, and the INTERTAN (Trigen INTERTAN™, Smith & Nephew, Memphis, TN, USA) was used on 118 patients.

The medical records showed that the patients were called for postoperative follow-up every six weeks for the first six months. In the following period, two follow-up controls were made six months apart, and later, annual controls took place. The data of the follow-up visits included physical examination and radiological assessment findings involving the evaluation of the union in terms of the presence of infection, loss of reduction, implant failure, avascular necrosis, and additional signs related to poor union formation, with the position data of the lag screw by using X-ray imaging.

The functionality was based on the functional assessment scale in Harris’ 1969 article and the Western Ontario and McMaster Universities Osteoarthritis Index (WOMAC). The study utilized the Turkish variant of the WOMAC self-scores [14]. Harris Hip Scoring system (HHS) was used for functional evaluation. According to the HHS, the scoring results were classified as follows: 90-100 points: excellent, 80-89 points: good, 70-79 points: fair, and <70 points: poor result [15].

### Preoperative management

All cases underwent the same preoperative preparation procedures: Following admission to the clinic, skin traction with a 2 Kg load was applied, and proximal tibial skeletal traction was applied on patients with displaced fractures, especially those with reverse oblique or subtrochanteric extension. Analgesia was provided with appropriate analgesics, and low molecular weight heparin was administered. Routine hematologic, biochemical, and microbiologic examinations were performed, and the patient was prepared for operation. An anesthesia consultation was conducted after the examinations were finalized. For patients with additional pathology detected in the tests, the relevant department consultation was requested, and the patient’s risk for surgery was determined. Elective operations were planned for patients with high risk. Two to four units of blood were prepared for each patient, considering additional orthopedic pathologies. Parenteral antibiotics (Cefazolin Sodium 1 gr) were administered prophylactically one hour prior to the operation.

The essential planning of the measurements of the femoral neck-shaft angle and the diameter of the femoral canal on the intact side were performed based on radiographic images for all patients.

### The detailed surgical technique

The surgical procedures for the fixation of intertrochanteric femoral fractures with the PFN-A and the INTERTAN are summarised in S1 File.

### Follow-up

Cefazolin sodium 1 gram was administered three times intravenously for 48 hours for postoperative infection prophylaxis. All patients wore antiembolism stockings for four weeks postoperatively. Low molecular weight heparin was continued postoperatively for four weeks to prevent deep vein thrombosis prophylaxis in all patients.

Patients in both groups were enrolled in a rehabilitation program beginning from the first postoperative day. Patients with stable trochanteric fractures (intact trochanter minor and medial cortical support) were mobilized with the help of a walker to push as much weight as tolerated. Quadriceps strengthening knee and hip periarticular muscle rehabilitation was initiated. Partial weight bearing was advised for patients with unstable trochanteric fractures using canes, crutches, or walkers for the first six weeks postoperatively and with full weight bearing after the 6th week, increasing with the occurrence of callus development. At this stage, exercises were initiated to strengthen the hip and knee muscles. Suture removal was conducted after the second postoperative week.

### Statistics

The Statistical Package for Social Sciences (SPSS) 17.0 program was used to analyze the study findings. Categorical measurements were summarized as numbers and percentages, and continuous measurements were summarized as mean and standard deviation (median and minimum-maximum where necessary). The chi-square test or Fisher’s test statistics were used to compare categorical variables. In comparisons of continuous measurements between groups, the Student t-test was used for parameters with normal distribution according to the number of variables and ANOVA for comparisons of more than two variables. The Mann-Whitney U test was used for parameters without normal distribution, and the Kruscal-Wallis test was used to compare more than two variables. The Kaplan-Meier method was used for the survival curve, and the Log-rank test was used to calculate survival differences between groups. The statistical significance level was set as 0.05 in all tests.

## Results

### Demographic findings, fracture types and surgery results

The records showed that 194 files were available for analysis. The mean age of the patients included in the study was 70.9±16.4 years; 100 patients (51.5%) were male, and 94 (48.5%) were female. The most frequent preoperative comorbidity was hypertension (32%), followed by type 2 Diabetes mellitus (23%).

Simple falls (*n* = 177, 91.2%) were the leading cause of trauma. However, other causes included were in-vehicle traffic accidents, out-of-vehicle traffic accidents, falls from height, and gunshot injuries. In terms of the distribution of fractures according to the AO classification, the most common fracture was 31A2 (52%), followed by 31A1 (27%) and 31A3 (21%).

Table 1 presents patients’ demographic and clinical characteristics, classified according to nail type.

**Table 1:** The demographic and clinical characteristics of the patients.

		PFN-A (*n* = 76)	INTERTAN (*n* = 118)
**Nail type**	Long	16	18
	Short	60	100
**Gender**	Male	39	60
	Female	37	58
**Type of trauma**	Simple fall	67	110
	Falls from height	3	1
	Out-of-vehicle traffic accidents	3	2
	In-vehicle traffic accidents	3	3
	Gunshot injuries	-	2
**Fracture side**	Right	33	62
	Left	43	56
**Fracture classification according to AO**	31A1	21	31
	31A2	38	63
	31A3	17	24
		**Mean±SD**	**Mean±SD**
**Age (year)**		65.6±17.7	74.6±14.2
**Time between fracture and surgery (day)**		5.38±3.8	4.05±2.6
**Hospitalization duration (day)**		8.96±4.3	8.73±3.9
**Duration of anesthesia (min)**		127.5±38.4	127.3±35.3
**Duration of surgery (min)**		95.8±29.9	90.3±25.1

Regarding the type of anesthesia, the majority of the patients were operated under general anesthesia (82%) with endotracheal intubation. Notably, there was a significant difference between the anesthesia durations based on the type of fractures. The duration of the anesthesia of the 31A1 fracture surgeries was significantly higher. In addition, despite a lack of significance, the duration of surgery was also prolonged in 31A1 cases (Table 2).

**Table 2:** The comparison of anesthesia and surgery durations in terms of fracture types and locations.

	Right Hip						Left Hip						
	31A1		31A2		31A3		31A1		31A2		31A3		
	*n* = 22		*n* = 54		*n* = 19		*n* = 30		*n* = 47		*n* = 22		
	Med	Min-Max	Med	Min-Max	Med	Min-Max	Med	Min-Max	Med	Min-Max	Med	Min-Max	*p*
Anesthesia (min)	120	80-180	120	75-230	150	80-205	110	60-240	120	35-255	142	90-255	**0.001**
Surgery (min)	92	45-120	90	60-210	115	50-160	80	40-225	85	20-255	105	65-220	0.053

Although postoperative blood transfusion amounts varied due to comorbidities and injuries, an average of one unit of blood was transfused in both groups.

Following the operation, 12 (3%) cases required intensive care unit follow-up.

### Survival analysis

**Fig 1:**
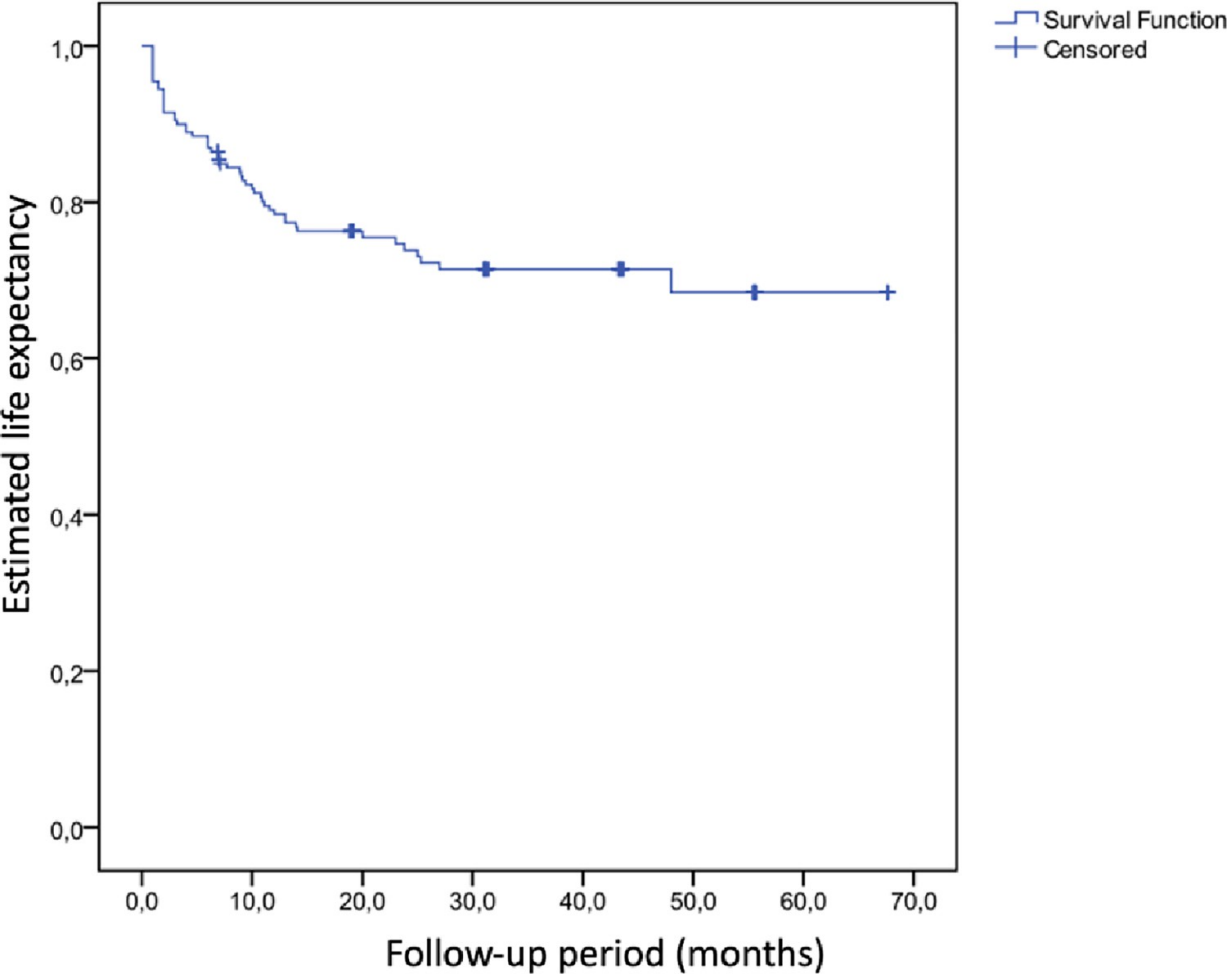
Survival analysis.

The survival analysis revealed that mortality rates, 12.5% in the first 1.5 months, 17.5% in the first six months, and 22.7% in the first year, increased to 26.5% in the long-term follow-up files after one year. According to the Kaplan-Meier method, the estimated survival of the patients included in the study was 50.3±2.1 (95% CI 46.3-54.4) months (Fig 1). The estimated one-year survival rate was 78.4%, the two-year survival rate was 73.0%, and the four-year survival rate was 68.4%. The type of nail selected had no statistically significant effect on survival (P = 0.581). Similarly, the fracture type had no statistically significant impact on survival (P = 0.400).

### Postoperative findings

The average follow-up periods of the patients in the PFN-A and the INTERTAN groups were 31.2±3 (1.0-60.0) and 19.1±5(1.0-60.0) months, respectively (P<0.001).

**Fig 2:**
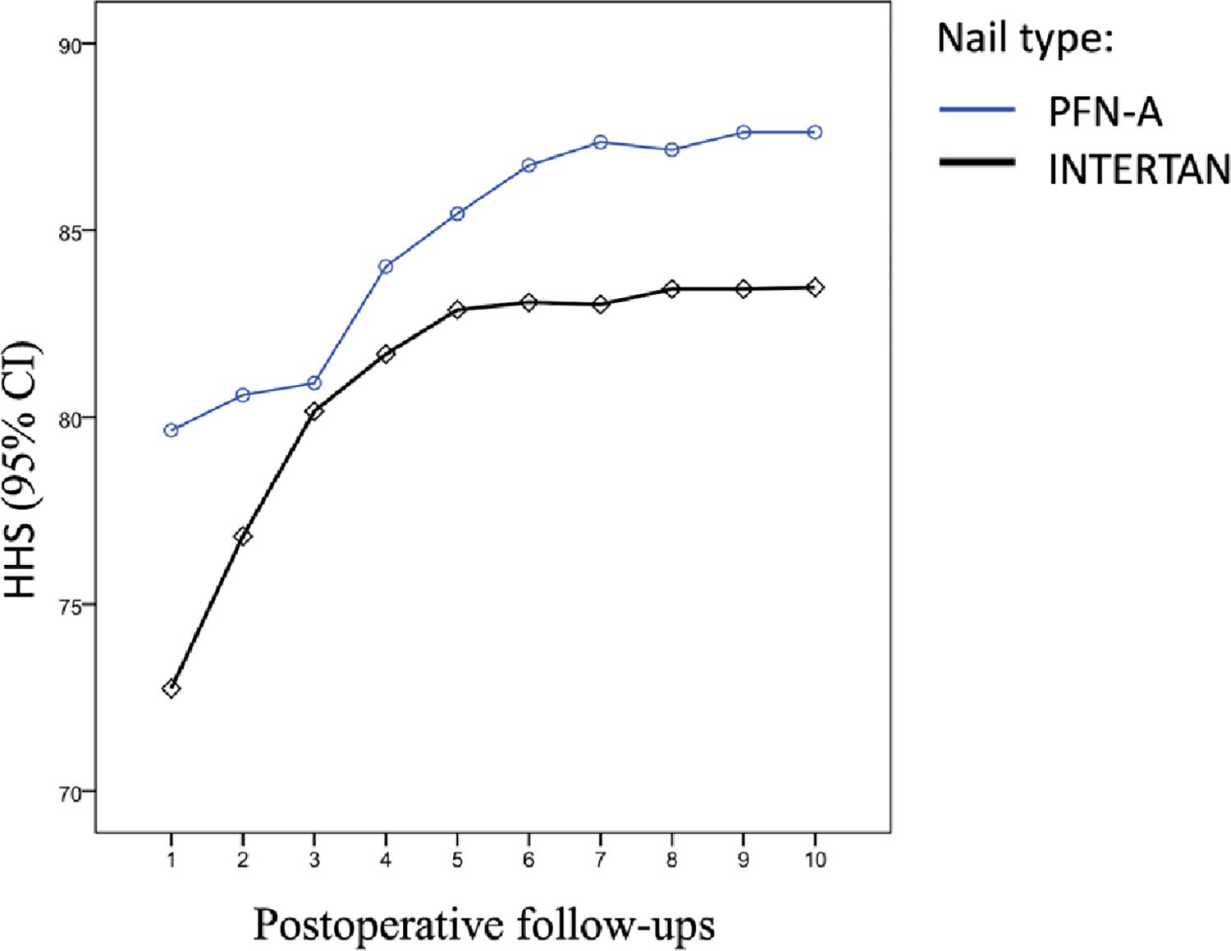
The mean Harris Hip Scores (HHS) values recorded at follow-up examinations.

The average HHS scores of the PFN-A and the INTERTAN groups were 85.3±14.2 and 82.8±14.0, respectively. According to the HHS scores assessed at the last follow-up visit, 7.7% of the patients had a poor score, 6.2% had a fair score, 66% had a good score, and 20.1% had an excellent score. Analysis of the mean Harris Hip Scores in both nail groups showed that only the mean scores at the 1st follow-up were statistically significantly different (P = 0.025) (Fig 2).

**Fig 3:**
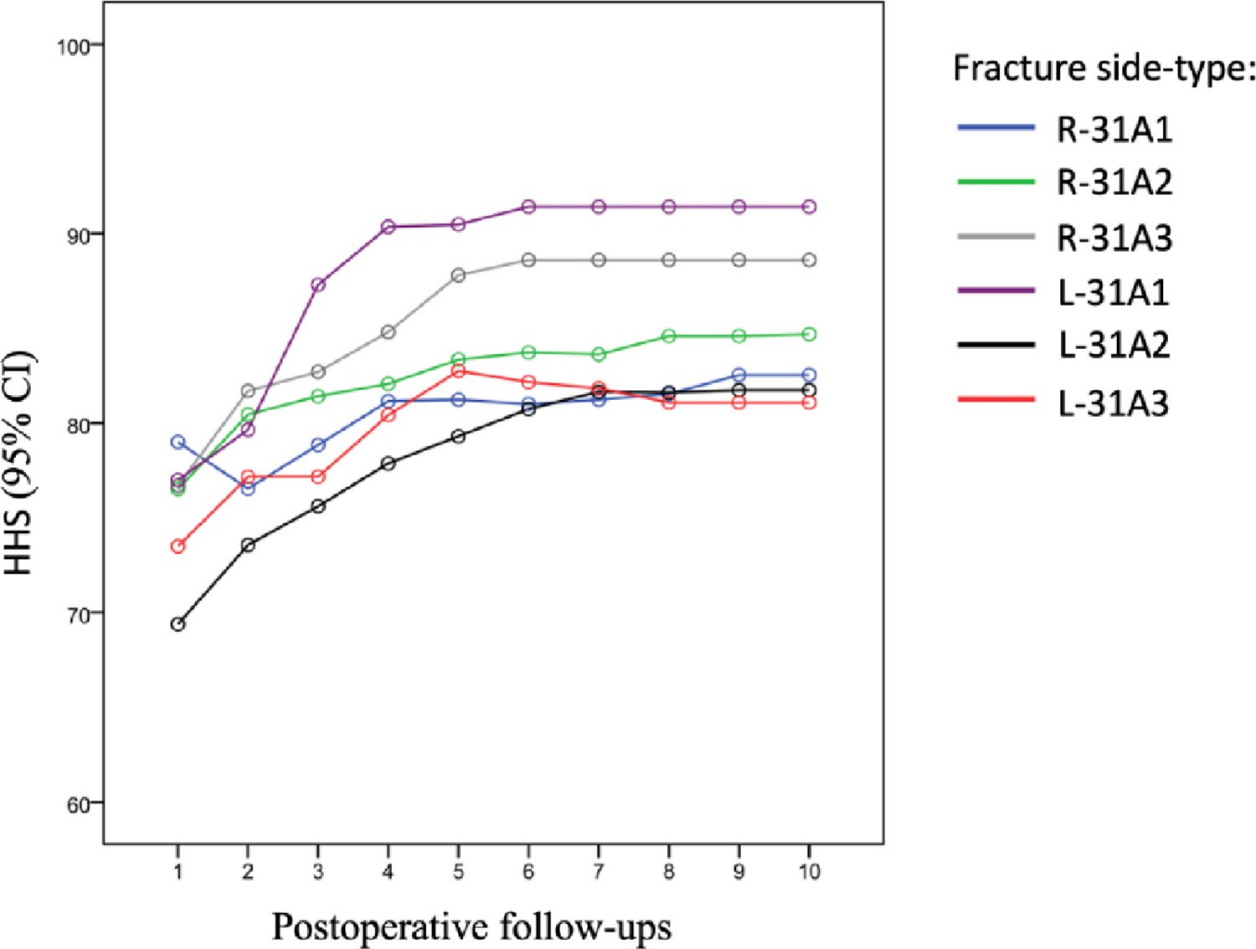
The mean Harris Hip Scores (HHS) values were categorized according to the type and the side on which they were located.

There were no differences regarding the comparison of the mean HHS values when separated according to the type and the side on which they were located (Fig 3).

The average WOMAC scoring for the PFN-A and the INTERTAN groups were 19(0-76) /96 and 21(0-72) /96, respectively, and the difference was insignificant.

There were eight cases requiring revision surgery. One PNA-A patient whose postoperative fracture reduction could not be performed properly with a long nail was fixed with a plate screw the next day. Another PNA-A patient had irritation at the distal screw locking site, and the distal screw was removed. There were three patients with implant cutting out of the fixation; two INTERTAN patients underwent total hip arthroplasty; blade removal was performed on the third patient with PFN-A.

Two PFN-A cases had deep infections, and the nails were removed. Finally, one INTERTAN patient had tensor fascia lata irritation, which was tenotomized. The incidence of avascular necrosis was not observed in any of the cases.

The mean Tip-Apex Distance measurements of the PFN-A and the INTERTAN groups were 17 (7-33)mm and 15 (4-30)mm, respectively. The average was 16 mm for all cases.

The patients were evaluated using the Cleveland Bosworth quadrants [16] to determine the position of the blade/lag screws sent from the femoral neck to the head, and 60.5% in the PFN-A group and 42.4% in the INTERTAN group were located in the exact center regions (Table 3).

**Table 3:** The distribution of blade/lag screws according to Cleveland-Bosworth quadrants.

Cleveland-Bosworth quadrants	PFN-A group	INTERTAN group
**5**	46/76 (60.5%)	50/118 (42.4%)
**2**	16/76 (21.1%)	44/118 (37.3%)
**Others**	14/76 (18.4%)	24/118 (20.3%)

There was no statistically significant difference between the fracture types of the patients included in the study in terms of age, time between fracture and surgery, length of hospitalization, duration of anesthesia and surgery, Tip-Apex distance, transfusion amount, WOMAC index, HHS, and follow-up period duration. However, the duration of surgery and, in parallel, the duration of anesthesia was longer in 31A3 fractures, an unstable fracture type. Also, in terms of fracture types, when the mean HHS at each follow-up time and the change in HHS over time were analyzed, the measurements were not statistically different.

## Discussion

The literature lacks a consensus on the gold standard treatment method in the surgery of proximal femur fractures, especially extracapsular hip fractures. Reports comparing the results of studies focusing on the fixation of extracapsular hip fractures in adults with intramedullary nails and extramedullary sliding hip plates indicate that no statistically significant difference was found in terms of blood loss, transfusion rates, complications of fracture fixation, postoperative complications, and hospital stay [11,17]. Our study results were in parallel with the findings reported in the studies and there were no statistically significant differences between the variables. Besides, many studies report that the majority of patients admitted with hip fractures were elderly, and 75% of them were female [18–21]. In our study, although the average age was in parallel with the literature, the ratio of females to males was 94/100, and we think that the higher proportion of male patients may be explained by the fact that elderly males in our society tend to spend more time outside the home.

Although recommended for use in fractured osteoporotic bones, there are reports indicating that approximately one in ten elderly patients expressed thigh pain complaints, which was attributed to biomechanical issues specific to PFN-A [22,23]. Concerning the statement, the average follow-up duration of the cases operated using PFN-A was found to be higher in the study. The increase in the duration might have been due to the patient’s desire to adhere to the follow-up schedule and express their complaints, but excluding the significance of the first follow-up HHS scores, there were no differences between the functional scores. Thus, the non-significant finding prevents us from establishing a difference between the two methods.

The studies focused on the types of trochanteric fractures patients surgically treated using nails, including the report of Aguado-Maestro et al., stated that around 50% were 31A2 and 31A3 ranked at the bottom with varying rates between 15% and 25% [24,25]. The study findings were similar regarding the rates of the 31A1, 31A2, and 31A3 types, with 27%, 52%, and 21%, respectively. The method of anesthesia varied considerably among the researchers regarding intramedullary nail treatment surgery [24,26–28]. The findings showed that 82% of the operations were performed under general anesthesia. One interesting finding of the study was that the duration of the anesthesia among the fracture types revealed that the duration of the 31A3 fractures was significantly increased (P<0.001). The literature search for studies comparing the anesthesia durations based on fracture types yielded no results. Nevertheless, the increased duration may be attributed to the complexity of the fractures requiring more meticulous work.

The studies show that the rates of cases requiring revision surgery for proximal femur nail operations varied between zero and 9% [24,26,27,29]. The reoperation rate in our study was 4.12%, which was within the range of the results reported. Nevertheless, the radiographic images of these cases after the first operation showed that the blades were appropriately positioned in the femoral head. In particular, the acetabular penetration due to the femoral head cut-out was believed to be related to the bone quality.

Baumgartner et al. stated in their studies that TAD values above 24 mm increased the risk of mechanical failure, and values below 24 mm amplified the risk of damage to the cartilage structure of the femoral head and acetabulum [30,31]. Our study’s TAD measurement values of PFN-A and INTERTAN groups were 17 (7-33) mm and 15 (4-30) mm, respectively. Studies comparing PFN-A and INTERTAN nails reported higher values ranging between 21 and 26.6, including the study of Sahin et al. indicating that more than 80% of the cases had values above 25 mm [24,28]. In our study, the TAD was shorter, suggesting a benefit in terms of mechanical stability. Moreover, no severe complications were observed in relation to the increased risk of damage to the cartilage structure of the hip joint.

Regarding blade positioning, 60% of the blades in the PFN-A group and 42% in the INTERTAN group, with a total average of 49%, were placed in the number 5 region, which is the center point according to the Cleveland Bosworth quadrants [16]. Quadrant placement rates in similar studies state that the positioning rates of the blades in quadrants number 2 and 5 were between 45% and 81% [24,32]. Regarding blade positions, our results were similar to the quadrant rates in the literature.

In our study, the radiographs of 3 patients who were cut out showed that blade tip placement was in 5 quadrants in 2 patients and in 2 quadrants in 1 patient.

Although there was no statistically significant difference between the measurements at other follow-ups, the mean HHS value of the PFN-A nail group at the 1st follow-up was higher than that of the INTERTAN group (P = 0.025). The average HHS scores of the PFN-A and the INTERTAN groups were 85.3±14.2 and 82.8±14.0, respectively. Mean HHS scores were calculated as poor in 7.7%, fair in 6.2%, good in 66%, and excellent in 20.1% of the cases. In similar studies in the literature, the mean HHS score was reported to be between 80 and 85 [21,27]. Sahin et al., in their studies using PFN-A, found that 13.3% of the patients reported poor, 20% were fair, 42.4% were good, and 24.4% had excellent HHS results [28]. In another study comparing PFN-A with INTERTAN, the HHS was 82.6±1.3 in the PFN-A group and 80.2±13.7 in the INTERTAN group [26]. Many of the abovementioned studies have failed to find any links between nail types and functional outcomes, similar to the findings of our study.

Kesmezacar et al. compared internal fixation with hemiarthroplasty in elderly patients with intertrochanteric femur fractures and reported that the mortality rate in the first six months was 48.8% in patients with endoprosthesis and 34.2% in patients with internal fixation. Since postoperative mortality rates were higher in the hemiarthroplasty group, they suggested that internal fixation should be considered as the first treatment option https://www.zotero.org/google-docs/?P9Wfet[33]. Kim et al. compared the results of hemiarthroplasty and internal fixation with PFN in unstable intertrochanteric fractures and reported mortality rates at three years as 55% in the hemiarthroplasty group and 17% in the PFN group [34]. In another PFN-A series, 1-year mortality was found to be 12.2% [28]. In our study, the mortality rate increased from 22.7% in the first year to 26.5% in the following year, and the 4-year life expectancy was 68.4%. The higher final mortality rate than those documented in the literature might be attributed to two important factors: the increase in the follow-up period and the average age of the study population. Firstly, the studies with longer follow-up durations reported higher rates, which was closer to the study results. Second, the elderly population in which the studies were conducted dies of other causes as the follow-up period increases, so the mortality rates increase dramatically as the follow-up period increases.

Moreover, the nail type was not associated with mortality rates, which was consistent with the results of other studies.

The retrospective design, the limited number of files included in the analysis, and having been conducted in a single center were the main limitations of the study. Additionally, bone mineral density examination results were not included in the analysis, which otherwise might have yielded different outcomes in the interpretation of the results. Nonetheless, the inclusion of radiological and functional assessment data and the long follow-up duration data have enhanced the value of the results.

## Conclusion

In conclusion, in the comparison of the two nail types in terms of complication rates and clinical data, neither of the nails has proven to be superior to the other. Nevertheless, surgeons might have to be aware of the prolonged anesthesia durations in 31A1 fracture operations. Moreover, the temporary difference in the first follow-up HHS scores should not be considered an indicator of the overall functional outcome because long-term follow-up HHS scores did not differ.

## Supporting information

S1 File “The surgical procedures for the fixation of intertrochanteric femoral fractures with the PFN-A and the INTERTAN” is the S1 File title. (PDF)
