# Supplementary material for: A retrospective comparative study of the clinical and radiological outcomes of intertrochanteric fractures treated with proximal femoral nail antirotation (PFN-A) and INTERTAN nail
Source: PLoS One. 2025 Jan 6;20(1):e0316954. doi: 10.1371/journal.pone.0316954 (PMC11703086; doi:10.1371/journal.pone.0316954)
Supplement: S1 File — (PDF) [file pone.0316954.s001.pdf]

The surgical procedures for the fixation of intertrochanteric femoral fractures with the PFN-A and the INTERTAN

The operations were performed under general (endotracheal intubation, laryngeal mask) or regional (spinal, spinal-epidural) anesthesia. After anesthesia procedures, patients were placed on the traction table in a supine position, with the upper extremity on the fractured side fixed above the trunk with an arm holder. The upper body was pulled approximately 10-15º toward the unaffected side for unobstructed access to the medullary canal. For displaced, unstable fractures, the abduction, external rotation, adduction, and internal rotation reduction maneuver described by Leadbetter for proximal femur fractures was applied [16]. The closed reduction maneuvers were performed for the fractures by controlling the reduction status under the guidance of a fluoroscopy device. The skin was cleaned in the surgical area after the reduction was found appropriate in AP and lateral planes by fluoroscopy device control. Then, the fracture side was stained with 10% povidone-iodine from the ankle to the chest level. After surgical draping, the surgical field was covered with a sterile wound dressing. The patient was prepared for fluoroscopy by wearing a sterile sheath. A 5 cm lateral longitudinal incision is made approximately 5 to 10 cm proximal to the tip of the greater trochanter. An incision parallel to the fibers of the gluteus medius was created and separated flush with the fibers of the gluteus medius. The 3.2 mm guidewire was applied from the apex of the greater trochanter or lateral to the greater trochanter to accommodate a mediolateral angle of 4 or 6 degrees because the mediolateral angle of the PFN-A nail is 6° and that of the INTERTAN nail is 4°. The guidewire was advanced 15 cm from the entry point into the medullary canal. The position of the guidewire, in terms of straightness and existence inside the medullary canal, was confirmed by images laterally. Following the confirmation on both planes on the fluoroscopy, the proximal femur was reamed to 7-8 cm with a 16 mm stop reamer using a tissue protector over the guidewire. During the drill guide and nail preparation phase, the protective guide and proximal femur reamer were placed at the trochanteric apex over a 3.2 mm guide wire in the medulla. Through the 3.2 mm guide wire, the proximal femur reamer was held with the T-arm universal holder and reamed until it rested against the protective guide. Then, the protective guide and guidewire were removed. The appropriate angle guide was connected to the nail, and the compatibility of the angle guide and the drill guide was checked outside. The system's accuracy was based on the alignment of the sheath apparatus sent through the proximal hole. The insertion of the appropriate diameter nail was performed by manually pushing the nail through

the trochanter major type by applying rotational forces without a hammer. The placement of the nail was checked using fluoroscopy in both planes. The nail is inserted to a depth that allows the blade or lag screw to be placed in the center of the femoral head. Using the targeting arm attached to the insertion handle, the correct depth of nail placement was verified in the anteroposterior (AP) view by placing a wire parallel to the guidewire track on the skin. The drill-sleeve assembly is advanced through the mini-incision from the targeting arm and soft tissues to the lateral cortex. The drill-sleeve guide was clicked and precisely placed on the femur lateral cortex. Using the targeting arm, a new guidewire was pushed through the protective guide. The AP and lateral images confirmed the central axis positioning of the guidewire on the femoral neck. The guidewire was advanced to the subchondral zone in the femoral neck, ensuring the wire tip was no closer than 5 mm to the joint.

In cases with the PFN-A, a step drill bit of size 11mm was advanced over the guidewire to the femur head, opening the lateral cortex, and fixed at the point verified according to the helical screw. After measuring the insertion depth of the guidewire using the ruler on the guidewire, the appropriate blade/screw length was determined. The helical screw was connected to the inserter, and a light counterclockwise force was applied. The helical screw was forced towards the femur head by hand over the guidewire and was inserted to the stop within 5–7 mm from the joint. If the fracture gap had to be closed following the lag screw's rotational locking, interfragmentary compression was carried out by rotating the compression nut in a clockwise direction prior to removing the drill sleeve and inserter handle. After enough interfragmentary compression, the locking mechanism in the upper part of the nail to lock the lag-screw rotation was tightened. A stab incision was made, and the drill sleeve and trocar were inserted through the selected locking hole in the aiming device. The femoral shaft was drilled, and a cortical screw was inserted in the appropriate length bi-cortically. The assembly was then removed, and the nail end cup was inserted.

In patients with the INTERTAN, after confirming the guide pin position in the femoral neck, the INTERTAN 7.0mm Compression Screw Starter Drill was inserted into the Lag Screw Drill Sleeve beneath the guide pin. The lateral cortex of the femur was drilled using the 7.0mm Compression Screw Starter Drill. A 7.0mm Compression Screw Drill was attached and inserted through the Lag Screw Drill Sleeve into the hole created by the Compression Screw Starter Drill. The Compression Screw Drill was advanced with the help of the fluoroscopy device; following the completion of the drilling process, the Anti-Rotation Bar was inserted beneath the guide pin. Then, over the guide pin using the Lag Screw Drill, the Lag Screw was inserted. The Anti-Rotation Bar beneath the guide pin was removed and replaced with the Compression

Screw. During the installation of the Compression Screw, traction was loosened simultaneously. After enough interfragmentary compression on the fracture site, the Integrated Interlocking Screws were locked by the locking mechanism on the upper part of the nail. The position of the screws was checked using the fluoroscopy. The protective guide and support nut are loosened and removed by pressing the button on the clamping part of the aiming arm. The protective guide for the distal locking screw was then placed through the hole on the aiming arm, and a pointer sleeve was inserted. A scalpel incision was made at the spot where the sleeve contacts the skin. The drill bit guide was attached to the bone through the locking hole on the aiming arm for distal locking, and the bone was drilled after removing the sleeve. The screw of the appropriate length was inserted after the drill guide was removed, and distal locking was achieved. The assembly was then removed, and the nail end cup was inserted.

Following the completion of the surgery of both methods, control images were taken in the anteroposterior and lateral planes, and the fixation status was assessed in the operating room. The surgical field was washed with 0.9% sodium chloride, and the incisions were closed after bleeding control.
